# Unraveling metagenomics through long-read sequencing: a comprehensive review

**DOI:** 10.1186/s12967-024-04917-1

**Published:** 2024-01-28

**Authors:** Chankyung Kim, Monnat Pongpanich, Thantrira Porntaveetus

**Affiliations:** 1 Center of Excellence in Genomics and Precision Dentistry, Department of Physiology, Faculty of Dentistry, Chulalongkorn University, Bangkok, Thailand; 2 Graduate Program in Bioinformatics and Computational Biology, Faculty of Science, Chulalongkorn University, Bangkok, Thailand; 3 Department of Mathematics and Computer Science, Faculty of Science, Chulalongkorn University, Bangkok, Thailand; 4 Center of Excellence for Cancer and Inflammation, Chulalongkorn University, Bangkok, Thailand; 5 Graduate Program in Geriatric and Special Patients Care, Faculty of Dentistry, Chulalongkorn University, Bangkok, Thailand

**Keywords:** HiFi, Microbiome, Nanopore microbes, ONT, Pacbio

## Abstract

The study of microbial communities has undergone significant advancements, starting from the initial use of 16S rRNA sequencing to the adoption of shotgun metagenomics. However, a new era has emerged with the advent of long-read sequencing (LRS), which offers substantial improvements over its predecessor, short-read sequencing (SRS). LRS produces reads that are several kilobases long, enabling researchers to obtain more complete and contiguous genomic information, characterize structural variations, and study epigenetic modifications. The current leaders in LRS technologies are Pacific Biotechnologies (PacBio) and Oxford Nanopore Technologies (ONT), each offering a distinct set of advantages. This review covers the workflow of long-read metagenomics sequencing, including sample preparation (sample collection, sample extraction, and library preparation), sequencing, processing (quality control, assembly, and binning), and analysis (taxonomic annotation and functional annotation). Each section provides a concise outline of the key concept of the methodology, presenting the original concept as well as how it is challenged or modified in the context of LRS. Additionally, the section introduces a range of tools that are compatible with LRS and can be utilized to execute the LRS process. This review aims to present the workflow of metagenomics, highlight the transformative impact of LRS, and provide researchers with a selection of tools suitable for this task.

## Introduction

The massive development in technology through the decades has allowed scientists to peer into the world of microbiome. In the human body, there are an estimated 10–100 trillion microbes that form a balance with the system [1]. Dysbiosis or an imbalance in the microbial population has been shown to be associated with disorders such as obesity, type I and II diabetes, autoimmune diseases, neurological conditions, and cancers [2]. The impact of microbes on human health has led to the development of metagenomics. Metagenomics is a scientific field focused on analyzing the genetic material of microorganisms within their natural habitats to acquire taxonomic and physiological insights [3]. This approach enables various applications such as assessing relative abundance, conducting taxonomic profiling, evaluating community richness, performing functional profiling, conducting pathway analysis, examining phylogeny, and detecting pathogens [4]. A proper understanding of human health and microbiome helps develop targeted therapeutic strategies. For example, metagenomic analysis can detect shifts in microbial abundance in response to interventions in inflammatory bowel disease, identify specific microbial signatures for potential intervention targets for type 2 diabetes mellitus, and better understand microbiome’s influence on immune function and the gut-brain axis to develop treatment for autoimmune diseases and neurological disorders [5, 6]. In summary, metagenomic analyses shed light on the importance of microbiome balance and inspire innovative strategies to combat health concerns.

**Fig. 1:**
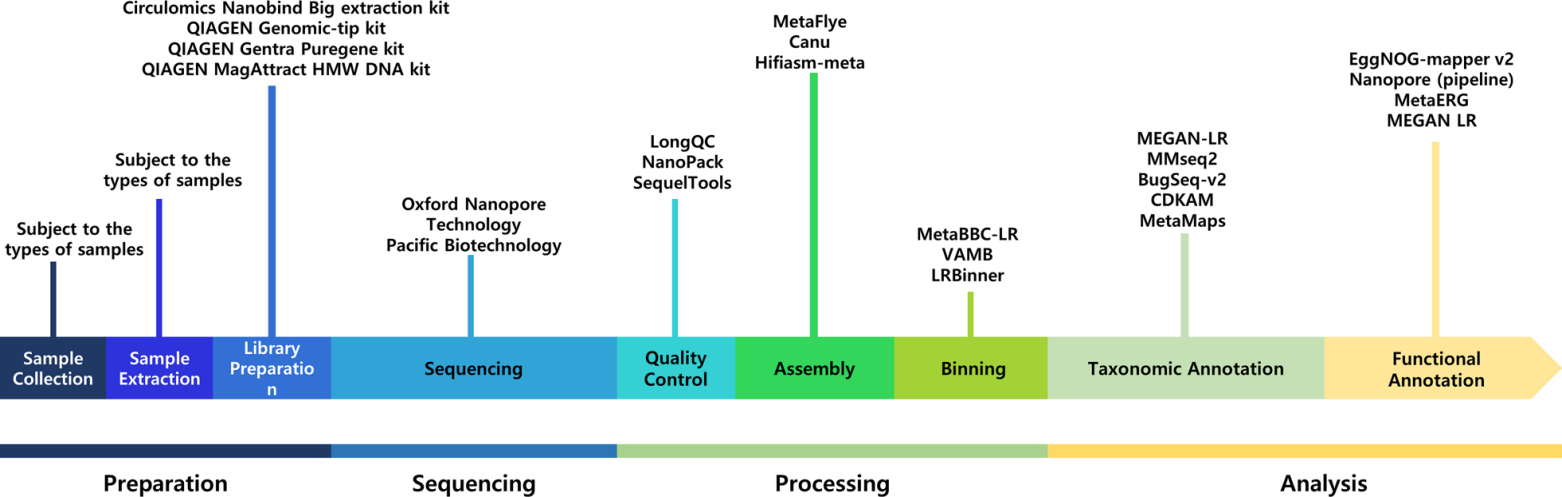
Pipeline of metagenomics analysis with long-read sequencing. The figure illustrates the step-by-step pipeline for metagenomics analysis beginning with sample collection, followed by sequencing, data processing, and analysis

Metagenomics process can be divided into four major steps: (1) sample preparation (sample collection, sample extraction, and library preparation), (2) sequencing, (3) processing (quality control, assembly, and binning), and (4) bioinformatics analysis (taxonomic annotation and functional annotation) (Fig. 1). Two major methods of metagenomic sequencing are 16S rRNA sequencing and shotgun metagenomic sequencing. Additionally, alternative markers such as the 18S rRNA gene or other protein-coding genes like gyrB are available options [7, 8].

## Long-read sequencing (LRS)

LRS is classified as third generation sequencing, being led by Pacific Bioscience (Pacbio) and Oxford Nanopore Technology (ONT) [9]. The length of the LRS can vary depending on the specific platform and technology used. The PacBio Sequel II system could produce reads with an average length of around 10–20 kilobases (kb), with some reads extending beyond 100 kb. On the other hand, ONT MinION and GridION platforms could generate reads with average lengths ranging from a few kilobases to over 100 kilobases, with maximum reads up to 2.273 metabases (Mb) in length [10]. LRS offers several advantages over SRS methods. Firstly, the longer reads result in fewer fragments, reducing the complexity of genome assembly and minimizing errors. Short-reads are often challenged when it comes to accurately assembling genomes, particularly in highly repetitive regions [11]. In contrast, the long-read length of over 10 kb in LRS enables the generation of fewer fragments with wider coverage, facilitating more efficient genome assembly. This advantage also enhances the detection of various types of variants, including large indels, complex rearrangements, structural variations, high GC content regions, and repetitive regions. In addition, epigenetics information such as 5-methylcytosine (5MC) is readily obtainable. Although LRS has previously faced criticism due to its relatively lower accuracy, recent advancements have significantly improved the accuracy rates. Advancements in nanopore sequencing encompass various aspects, with flow cells playing a crucial role. These cells house essential components for nanopore sequencing, including nanopore sensors, motor proteins, and other associated chemicals. Flow cells have undergone several iterations, with the latest being the R10.4 flow cell. This iteration represents a significant improvement in sequencing accuracy, speed, and the ability to handle larger sets of reads [12]. Traditionally, the major downside of LRS has been its high error rate when compared to SRS. The error rate in ONT arises from the challenge of controlling the speed of DNA molecules passing through the nanopore, while PacBio contends with random errors [13]. Despite these challenges, there have been improvements in accuracy. PacBio implemented circular consensus sequencing, elevating their accuracy to 99.8%, while ONT introduced new flow cells, achieving an improved accuracy of 99.5% [14]. Additionally, the integration of phi29 DNA polymerase has played a role in slowing DNA translocation and further reducing the error rate in ONT [15]. Although these accuracies still fall slightly short compared to SRS, PacBio's latest LRS technology iteration, Revio, has reached an impressive accuracy level of 99.9%, placing it on par with SRS [16].

The improved accuracy rates of LRS have enhanced its utility in various genomic analyses. However, LRS does have a notable drawback when compared to SRS due to its relatively higher sequencing cost. Despite the ongoing reductions in sequencing costs for both methods, LRS continues to be the more expensive option. The longer read lengths and more intricate sequencing technologies utilized in LRS contribute to its higher cost compared to SRS. While advancements and economies of scale may lead to future cost reductions in LRS, it currently remains a factor that researchers must consider when choosing between sequencing methods, considering their specific requirements and budget limitations.

## Sample preparation

### Sample collection

According to the National Institute of Health (NIH), metagenomics is “the study of the structure and function of entire nucleotide sequences isolated and analyzed from all the organisms (typically microbes) in a bulk sample.” Naturally, metagenomics allows researchers to study the community of microorganisms taken directly from natural habitats. Common samples of metagenomics include soil, water, feces, and saliva. Biological samples are usually frozen at −80C to prevent alteration in the microbial landscape, while environmental samples are frozen at different temperatures (− 20C to − 80C).

### Sample extraction

The development of LRS platforms has shifted the limitation from technology to the quality and length of DNA input. With LRS, the extraction must be pure and of high molecular weight. For metagenomics, any damage to the DNA or contamination can result in poor performance, lower read lengths, and even affect the library preparation step.

There are several factors that ensure the read length and quality of the sample: the genetic material within the microbial sample (1) is double stranded (except when dealing with viruses), (2) has not undergone multiple freeze thaw cycles, (3) has not been exposed to high temperature or extreme pH, (4) has no RNA contamination, (5) has no exposure to intercalating fluorescent dyes or UV radiation, and (6) does not contain denaturants, detergents, or chelating agents [17–19]. The extraction can be done manually or using commercial kits, depending on laboratories, types of sample, and experimental designs. When using a DNA extraction kit, it is important that the kit does not shear the DNA to below 50 kb, which is unsuitable for LRS. Some of the recommended kits are Circulomics Nanobind Big extraction kit (PacBio), QIAGEN Genomic-tip kit, QIAGEN Gentra Puregene kit, and QIAGEN MagAttract HMW DNA kit. The resulting DNA extraction should reflect the microbial community within the sample and contain adequate nucleic acids for library preparation and sequencing [19].

### Library preparation

Library preparation is a process where the nucleic acids are isolated, fragmented, end repaired, and linked to adapters via either tagmentation or ligation method. The DNA sample that is sheared too short during library preparation can be unsuitable for LRS [20]. There are specific library preparation kits available, such as ONT DNA by ligation, ONT Rapid library prep, and ONT 16S library prep. Library preparation for LRS requires reagents to be pipetted slowly to minimize shearing. This is a time-consuming process that can result in inconsistent read lengths, and even a small shift in pipetting volume can cause DNA shearing [20].

Different preparation kits require different amounts of minimum quantity and minimum concentration of the sample. For MinION, the genomic DNA is sheared by g-tubes to > 8 kb in length per fragment [21]. The fragments are end repaired and a non-templated dAMP is added to the 3’end of DNA fragment using dA-Tailing Kit. Protein-conjugated MinION adapters are ligated, and a tether protein is added to guide the tethered DNA molecule to the nanopore. Finally, the library is conditioned before being loaded onto the MinION sequencer [21]. Library preparation for PacBio is called SMRTBELL library preparation and starts with the generation of acceptable DNA fragments. The fragments can originate from either random shearing or the amplification of a region of interest. The process of creating the library involves ligating universal hairpin adapters to both ends of the fragment.

## Sequencing

### Sequencing platforms

Oxford Nanopore Technology (ONT) and Pacific Biosciences (PacBio) are two leading companies in the field of long-read sequencing (LRS), despite their shared goal, their procedures and technologies are distinct. In this section, mechanisms behind each platform are explored, as well as the advantages and limitations of each method.

### Oxford nanopore technology

Three ONT platforms are available for LRS. MinION, first released in 2016, operates 1 flow cell and has a maximum run time of 72 h, maximum yield of 40 to 50 Gb, and up to 512 available channels. GridION, released in 2017, operates 5 flow cells and has a maximum run time of 72 h, maximum yield of 200 to 250 Gb, and up to 2560 available channels. The last addition is PromethION, released in 2018, operates 48 flow cells, has a maximum run time of 68 h, a maximum yield of 8.6 to 15 Tb, and up to 144,000 available channels [22]. ONT platforms operate in a similar manner, for example, GridION is in essence five MinIONs.

In nanopore sequencing, a constant electric field is applied, leading to the observation of an electric current due to the presence of an electrolytic solution within the nanopore system. [23]. The density of the electric current is influenced by both the dimensions of the nanopore, which is a pore created from protein or synthetic material on a membrane, and the genetic material composition of the extracted DNA/RNA as it passes through the nanopore [24]. In order to sequence a strand of DNA or RNA, a sequencing adapter, which is a piece of DNA with an enzyme motor, is added to the sample [15]. After applying a constant electric field to the nanopore membrane, the sample strand translocates through the nanopore until the sequencing adapter reaches the top of the nanopore. The adapter then functions as a helicase, unwinding DNA and allows single strands of nucleotide to pass through the nanopore. When a strand of DNA or RNA passes through the pores (assuming α-hemolysin is used), which are covalently bound to cyclodextrin ((6-deoxy-6-amino)-6-n-mono(2-pyridyl)dithiopropanoyl-b-cyclodextrin) for increased selectivity, an electrical signal occurs, resulting in changes to the ion current. Each nucleotide type must have the ability to impede the ion flow into the pore for varying time intervals. By detecting the fluctuations in ion current caused by these blockages, the sequence of the strand being used can be identified.

### PacBio technology

PacBio offers five LRS platforms, but the RS II, released in 2013, has been overshadowed by newer platforms. The Sequel system, introduced in 2015, supports SMRT Cell 1 M, yielding up to 500,000 HiFi reads with over 99% accuracy, and offers up to 20 h of sequencing runtime per SMRT Cell. The Sequel II system, launched in 2019, supports SMRT Cell 8 M, capable of generating up to 4,000,000 HiFi reads with > 99% accuracy and provides up to 30 h of sequencing runtime per SMRT cell. The latest model in the Sequel system, the Sequel IIe, was released in 2021 with similar specifications to the Sequel II platform, but it is optimized for generating highly accurate HiFi reads. As of late 2022, the current newest model is Revio, which offers significant improvements over previous platforms. It enhances the SMRT cell design and computation capabilities, leading to increased throughput and reduced costs. Leveraging HiFi technology, Revio achieves higher accuracy in sequencing. Additionally, Revio is capable of direct methylation detection, making it a versatile and advanced tool for long-read sequencing applications. Despite having a slightly shorter read length of 15-18 kb, when compared to ONT's 10-100 kb, Revio compensates with its impressive high accuracy of 99.95%. It also offers shorter run times, taking only 24 h to complete a sequencing run. Additionally, Revio is capable of accurately detecting indels, making it a powerful and accurate tool for various long-read sequencing applications. According to the data release from PacBio, the advancements in processing time and cost efficiency with Revio enable up to 1,300 human whole genome sequencing to be performed annually at a price of less than $1000 per genome. Compared to Sequel IIe, Revio utilizes 25 million Zero Mode Waveguides (ZMW) instead of 8 million ZMW, resulting in increased throughput. Additionally, Revio offers a shorter run time of 24 h compared to 30 h in Sequel IIe, and it can produce a significantly higher data output of 360 Gb/day as opposed to 24 Gb/day. These improvements position Revio as a game-changer in the field of long-read sequencing, offering higher efficiency and lower costs for genomic research and analysis.

**Fig. 2:**
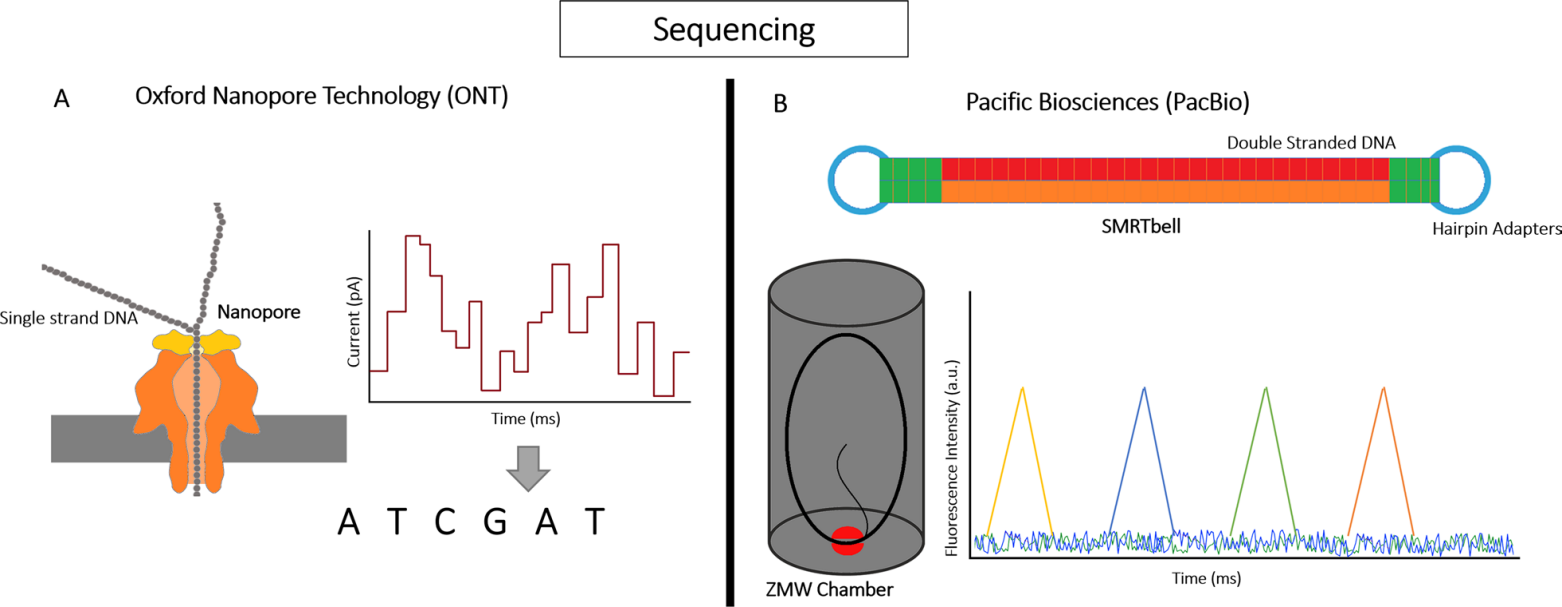
Overview of sequencing functional principle. **A** The ONT sequencing process initiates by passing a DNA or RNA strand through a nanopore—a small protein opening embedded in an electrically resistant membrane, functioning akin to a biosensor. A constant voltage applied to the electrolytic solution induces an ionic current through the nanopore. As a negatively charged DNA or RNA strand traverses the nanopore, inducing a shift from negative cis to positive trans, the motor protein influences the translocation speed. The change in ionic current resulting from this charge shift corresponds to the nucleotide sequence, enabling the identification of DNA/RNA strand bases. **B** The PacBio sequencing process involves fragmenting target double-stranded DNA molecules and ligating them to hairpin adapters, forming a SMRTbell—a closed, single-stranded circular DNA template. These SMRTbells are loaded onto a SMRT cell equipped with Zero-Mode Waveguides (ZMWs). A single polymerase binds to hairpin adaptors situated at the ZMW's bottom, initiating replication. To identify bases, four fluorescent-labeled nucleotides with distinct emission spectrums are introduced, producing a unique light pulse when incorporated into the polymerase. This series of light pulses is recorded and utilized to interpret the sequence of bases

Single molecule real time sequencing (SMRT) is a sequencing technology developed by PacBio. Similar to nanopore sequencing, SMRT requires a specific library preparation method. The library is prepared from approximately 5 mg of double-stranded DNA, which serves as the starting material for the SMRT sequencing process [25]. Next, hairpin adapters are ligated to the DNA molecules, creating SMRTbell, a circular structure that contains DNA inserts flanked by two hairpin adapters [26]. Once the primer and polymerase are annealed to the adapter in the library, it can be loaded onto the SMRT cell, which contains observation chambers known as Zero Mode Waveguides (ZMWs). The number of ZMWs in the SMRT cell can vary depending on the specific platform being used, ranging from 150,000 to a million or more. These ZMWs are crucial for single molecule sequencing, as they allow individual DNA molecules to be isolated and sequenced in parallel, resulting in the production of long-reads during the SMRT sequencing process [25]. SMRTbell, now polymerase bound, are loaded onto ZMWs, and multiple ZMWs are loaded onto one SMRTbell to maximize the throughput and read lengths [27]. The small diameter of ZMW allows only a minute volume for light detection. The fragments of DNA are pulled down to the bottom of ZMWs. Once the polymerase is bound to SMRTbell, the incorporated fluorescent labeled nucleotides start emitting fluorescent signals, which are recorded in real time [25]. Besides registering fluorescent colors, the time interval between nucleotides is measured in a process called interpulse duration (IPD). The signals are then converted into continuous long-reads (CLR) [28]. CLR are then converted into multiple reads or subreads, which is an individual sequence reads, via the removal of adapter sequences. The resulting subreads are combined to generate a single highly accurate consensus sequence called circular consensus sequence (CCS) [29]. A comparison of the sequencing technologies of ONT and PacBio can be found in Fig. 2, illustrating the key differences between nanopore sequencing and single molecule real-time (SMRT) sequencing.

## Processing

### Quality control phase

The quality control phase plays a critical role in filtering out unwanted and misleading data, particularly for traditional short-read techniques. In short-reads, various criteria are examined, including read length, GC content, quality score, sequence complexity distributions, sequence duplication, ambiguous bases, artifacts, and contaminations [30]. This meticulous assessment ensures that only high-quality and reliable data is retained for further analysis and interpretation. Software tools like FastQC are designed to take FASTA/FASTQ files as input and provide comprehensive analysis and quality assessment for sequencing data. FastQC displays essential statistics such as sequence quality, sequence content, GC content, N content (ambiguous bases), length distribution, and sequence duplication levels [31]. It also identifies overrepresented sequences and analyzes kmer content, allowing researchers to evaluate the overall quality and potential issues in their sequencing data. These functionalities aid in identifying potential problems, and guiding researchers in making informed decisions during downstream analysis and interpretation. After quality control, the sequence data can be filtered based on length, GC content, quality scores, number of sequences, and other criteria [30]. The sequence can be trimmed to a specific length. To avoid potential duplication issues, trimming should be performed before the filtering process [32]. This ensures that the filtered dataset retains the most relevant and non-redundant information, contributing to accurate downstream analyses.

LRS requires different quality control approaches than short-read sequencing. Some researchers use a hybrid method, sequencing the same sample with both short- and long-read technologies, to enhance accuracy and gain comprehensive insights into complex genomes and structural variants [33]. Unlike short-read QC, which requires trimming of the reads, long-read QC can only display visual output, while leaving filtering and trimming optional. Due to the inherent differences in long-read data provided by the two companies, a single quality control tool may not effectively cover both datasets. For instance, PacBio’s long-read data from the Sequel sequencing platform may lack meaningful Phred scores, which are available in ONT's data [34]. Consequently, specific quality control tools tailored to each platform are necessary to ensure accurate assessment and processing of the respective long-read datasets.

There are several quality control software tools available for handling long-read sequencing data. One such tool is LongQC, which is specifically designed for nanopore and SMRT sequencing quality control. LongQC offers a user-friendly interface and incorporates multiple modules and algorithms for comprehensive analysis. The coverage module in LongQC is a key feature that provides essential information such as general statistics, read length analysis, quality score assessment, coverage analysis, GC content analysis, error estimation, and more [34].

NanoPack is a widely used quality control tool for long-read sequencing (LRS) data. It includes several sub-tools, such as NanoQC, NanoPlot, Cramio, and more, providing a comprehensive suite for quality assessment and data analysis [35]. While NanoPack can be utilized for both ONT and PacBio data, the lack of Phred scores in PacBio data can limit its usage for certain quality control aspects. Phred scores are crucial for assessing base call accuracy and the quality of individual bases in the sequencing reads. Without Phred scores in PacBio data, some specific quality control metrics may not be as robustly evaluated in comparison to ONT data [34]. Nanopack2 serves as the successor to NanoPack, featuring various enhancements across its modules. Improvements include code optimization, increased plot generation options, and dynamic HTML plots within NanoPlot and NanoComp. Furthermore, Nanopack2 combines NanoFlit and NanoLyse functionalities into the Chopper tool, enabling comprehensive filtering based on read score, read length, contamination level, and other factors. In the latest version of Nanopack, Cramino, built on rust-htslib, has replaced the slower NanoStat to efficiently generate summaries for long-read sequencing experiments. Along with this major update, several other smaller improvements and changes have been implemented throughout the system [36]. Furthermore, Nanopack now offers the added functionality of filtering and trimming reads, providing users with the option to refine and process their LRS data based on specific criteria and quality thresholds. These updates make Nanopack a more robust and user-friendly tool for quality control, analysis, and data manipulation in LRS experiments.

SMRT Link is a web-based tool of PacBio that is compatible with all Sequel systems as well as the new revio system. It is a comprehensive tool specifically designed to work with PacBio data, making it one of the best choices for handling PacBio sequencing data. SMRT Link provides a wide range of functionalities, including sample setup, run design, run QC, data management, and SMRT analysis [37]. One of the key features of SMRT Link is its quality control function, which empowers users to efficiently sort, search, and filter the reads based on various parameters. These parameters include minimum and maximum subread length, minimum number of passes, minimum predicted accuracy, minimum read score, and others [38]. During the quality control phase, SMRT Link generates various informative data, such as sample information, run settings, total bases (in Gb), unique molecular yield (in Gb), productivity percentage, number of reads, and control information. Among the successfully quality controlled data, the plots such as polymerase read length, longest subread length, control polymerase RL, control concordance, base yield density, read length density, HiFi read length distribution, read quality distribution, and read length vs predicted accuracy can be viewed. Other tools such as SequelTools can perform quality control, read subsampling, and read filtering. The generated standard metrics includes N50, read length, count statistics, PSR (polymerase-to-subread ratio), and ZOR (ZMW-occupancy-ratio) [37]. However, SequelTools works primarily with CLR, rather than directly working with CCS which has higher accuracy. In general, both ONT and PacBio provide customized tools to efficiently handle the distinct data generated by their respective sequencing platforms. If researchers are analyzing data exclusively from a specific platform, it is advisable to utilize NanoPack2 for ONT data or SMRT Link for PacBio data. However, in the case of hybrid data usage where filtering or trimming is unnecessary, longQC is recommended as a suitable tool for managing the combined nanopore and SMRT sequencing datasets.

### Assembly

**Fig. 3:**
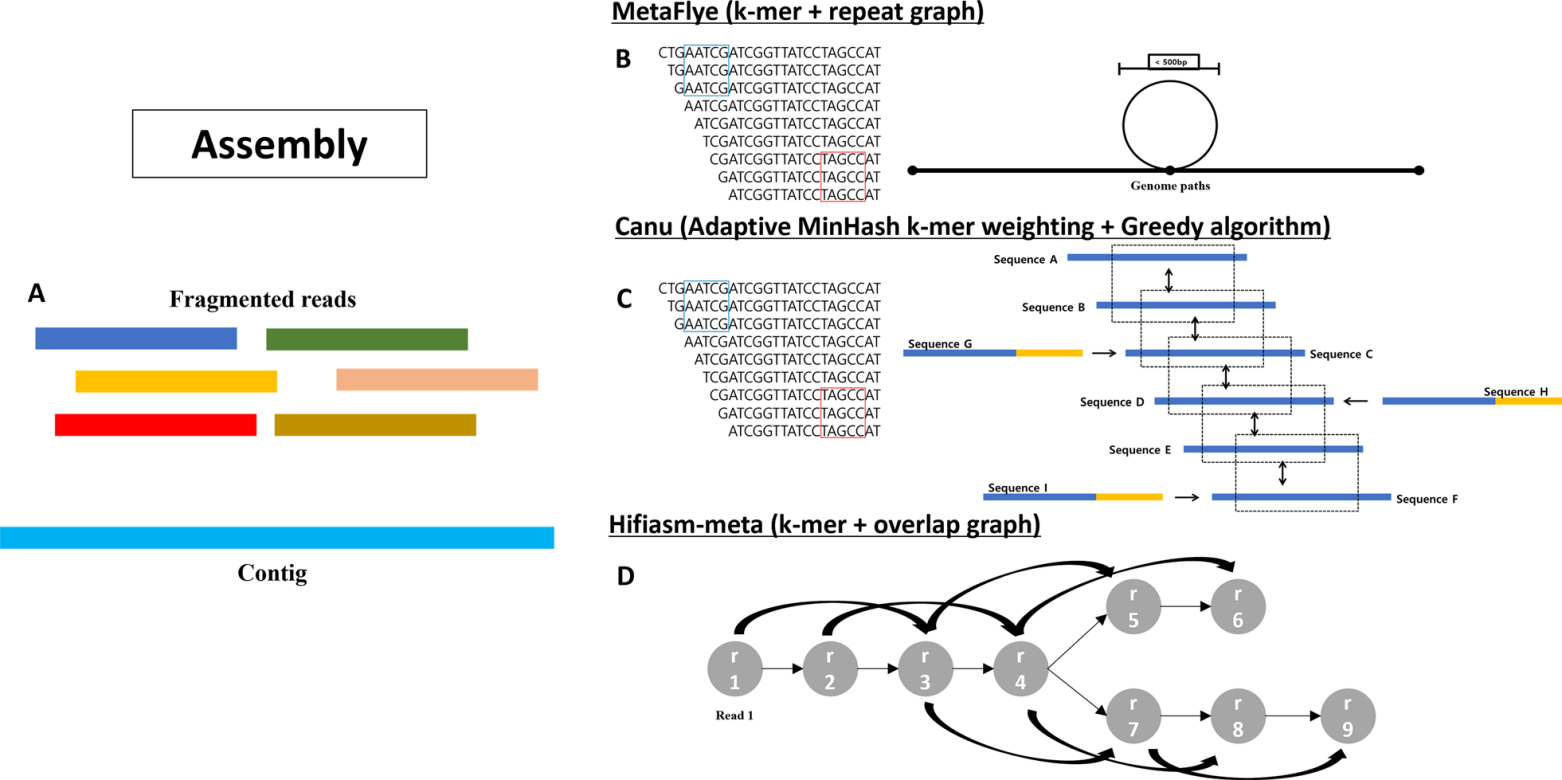
Overview of assembly algorithm. **A** Fragmented reads are overlaid and merged to reconstruct microbial genomes, with the longer reads enhancing the connections between each fragment. **B** MetaFlye, a LRS metagenome assembler, uses k-mer and repeat detection algorithm, which is particularly useful when detecting repeats inside a bubble. **C** Canu utilizes a form of k-mer algorithm known as adaptive MinHash k-mer weighting, in combination with an altered version of the greedy best overlap graph (BOG) algorithm. The greedy BOG algorithm, initially developed by Miller and colleagues, serves as the foundation for constructing the graph in Canu. Sequences with mutual best overlap are indicated with arrows going both ways. However, sequences G, H, and I have one-sided arrows, meaning that the best overlap regions are not mutual and are not included as part of the best overlap. **D** Hifiasm-meta utilizes k-mers to query reads and construct an overlap graph while retaining reads with rare k-mers, which typically correspond to low abundance sequences

An assembly is the process of merging fragmented reads obtained through sequencing to reconstruct the original sequence (Fig. 3A), and there are several approaches to perform it using LRS data. Two prominent methods for LRS assembly are HiFi assembly, which is designed specifically for LRS, and the hybrid method that combines HiFi sequencing with SRS methods. However, unlike genomics which deals with one organism, the challenge of metagenomics is to piece together thousands or millions of organisms simultaneously. The three most popular algorithms for genomic assembly are the greedy algorithm, overlap layout consensus (OLC), and De-Brujin graph methods [39]. The greedy algorithm overlaps each read into contigs and stops when no more reads or contigs can be joined. OLC involves calculating pairwise overlaps among all reads and constructing an overlap graph with nodes representing individual reads and edges denoting the overlaps. Each pathway through the graph results in a layout, and the most likely sequence is inferred through multiple alignments of the reads during the consensus stage [40]. In De-Brujin graph method, k-mer algorithm, which is an overlapping of small segments, to construct a graph based on the co-occurrence of k-mers across the reads [39].

Genomic assembly in metagenomics is challenging due to the vast number of organisms present in the sample. The metagenomic sample contains a diverse range of species with varying abundance levels, resulting in uneven read coverage across the genomes [41]. Also the low coverage of most species in a metagenomic sample compared to that of cultivated sample, can result fragmented and inaccurate metagenomic assemblies [41].

Traditional coverage statistics are rendered unreliable due to the uneven and often unknown representation of the variety of microbes within the sample. For example, unrelated individuals from different species with similar genomes may have a high identity rate, while closely related individuals from identical species with small genetic differences leading to lower identity rate. Both can complicate the assembly process [42]. This issue poses a serious problem of incomplete and fragmented assembly or even a misassembly when the difference in strains disrupts assemblers from resolving path across the assembly graph, such as OLC and de Brujin. The problem is partially resolved by the detection and masking of the genetic variation amongst closely related strains to generate consensus assembly [41]. However, the consensus assembly can result in annotation artifacts and the loss of individual strain information [39]. An uneven sequencing depth can lead to one organism receiving high depths of coverage, resulting in quadratic growth in computing time for the OLC algorithm and exacerbate the effects of errors in the De-Brujin graph algorithm [41]. Moreover, a large amount of input data is required to reconstruct rare strains, which requires a very high coverage depth that can significantly increase the computation cost [39]. It is important to understand the type of results that an assembler produces and to note that different assemblers can have very different performances. Metagenome assemblers produce contigs and assembly graphs and perform finding path across assembly graph and between contigs. Metagenomic assembly process can also create metagenome assembled genome (MAG), which refers to the collection of microbial genomes or scaffolds that share similar characteristics. MAGs are particularly useful when there is not enough high-quality reference genome [43].

Usually, metagenome assemblies use short-reads with fragments of ~ 1000 bp in length [44], which result in low repeat resolution and unresolved repeats. However, the recent advances in LRS technologies and the extraction technique for DNA with high molecular weight, enabled sequencing of long metagenomes [45]. LRS technology has improved the extraction of high molecular weight (HMW) DNA through various enhancements. These include minimizing DNA shearing during extraction, employing a magnetic bead-based method, and avoiding vigorous vortexing that could potentially fragment the DNA. The technology also enables the direct sequencing of single DNA molecules without the necessity for amplification and implements advanced library preparation protocols tailored for HMW DNA. These protocols involve gentle DNA extraction methods, steering clear of harsh conditions that could lead to DNA fragmentation [46]. LRS has the potential to improve metagenomic assemblies and overcome many limitations stated above. A few popular choices are metaFlye, Canu, and hifiasm-meta. MetaFlye is a long-read metagenomic assembler, modeled after Flye, as a fast long-read genome assembler. The original Flye attempts to estimate the set of genomic k-mers by selecting high frequency k-mers (solid k-mer) [47]. The metaFlye, on the other hand, favors high abundance species while low abundance species having lower solid k-mers are not assembled. Instead of relying on solid k-mer (high frequency), metaFlye uses a combination of global k-mer counting with local k-mer distribution analysis [45]. The new algorithm detects repeat edges in assembly graphs, which allows them to bypass or identify repeat regions within simple bubbles, superbubble, and roundabout. This allows metaFlye to not require structures such as superbubble to be acyclic, unlike many assemblers, and allowing the repeats within the bubble to be analyzed. This repeat detection algorithm uses iterative detection to go through all edges and categorize some edges as either repetitive or unique and grant strong resilience against read coverage with high nonuniform distribution [47]. A simplistic visual representation of the k-mer and an example of a bubble is shown in Fig. 3B.

Canu, a successor to Celera Assembler, is a reliable tool that can handle diploid, polyploid, and metagenomic assembly. Canu utilizes a new overlapping and assembly algorithm, which incorporates a tf-idf weighted MinHash based adaptive overlapping strategy and sparse assembly graph construction to improve assembly continuity with a decrease in the required depth of coverage and run time. The adaptive MinHash k-mer weighting allows the number of repeats to be for overlapping, while not eliminating the chances entirely. This is achieved by using MinHash Alignment Process to compare compressed sketches instead of comparing each individual k-mers for potential read overlaps, allowing Canu to compare entire reads to compressed sketches. The construction of sparse overlap graph uses a variation of greedy algorithm of best overlap graph. By only loading the best overlaps per read end, this method is memory efficient and works best with longer read lengths [48]. The basic concept of greedy best overlap graph, or BOG, is demonstrated in Fig. 3C. It was first adopted for Celera Assembler and was successful in producing one of the longest contigs among assemblers back when it was tested.

Hifiasm-meta, a tool developed in 2022, is designed to leverage the significantly enhanced quality of long-read sequences. The workflow of hifiasm-meta includes read selection, sequencing error correction, read overlapping, string graph construction, and graph cleaning. Going through 2000 reads per round, k-mer counts are recorded onto an empty hash table. For each read encountered, canonical k-mer are queried in the hash table for frequency. The percentiles 3%, 5%, and 10% percentiles are compared to the respective threshold values, and only the reads that do not reach the thresholds are kept. This allows the reads with rare k-mer to be kept. Hifiasm-meta keeps reads that have no overlapping reads in the middle section but have 5 or fewer overlaps on either end of the read. This ensures that the genome of low abundance is not discarded. Hifiasm-meta retains contained reads (reads contained within a longer read) if the identical overlapping reads are from different haplotypes and drops contained reads only if there is no similar haplotype nearby. Finally, at the graph construction, any overlaps between unitigs (a hifiasm terminology: a high confidence contig) from different haplotypes are rejected [49]. This process adds benefit of fixing assembly gaps by patching up unitigs. Unitig coverage is then used to filter overlaps. However, all these are applied to unitigs longer than 100 kb [49] (Fig. 3D).

### Binning

**Fig. 4:**
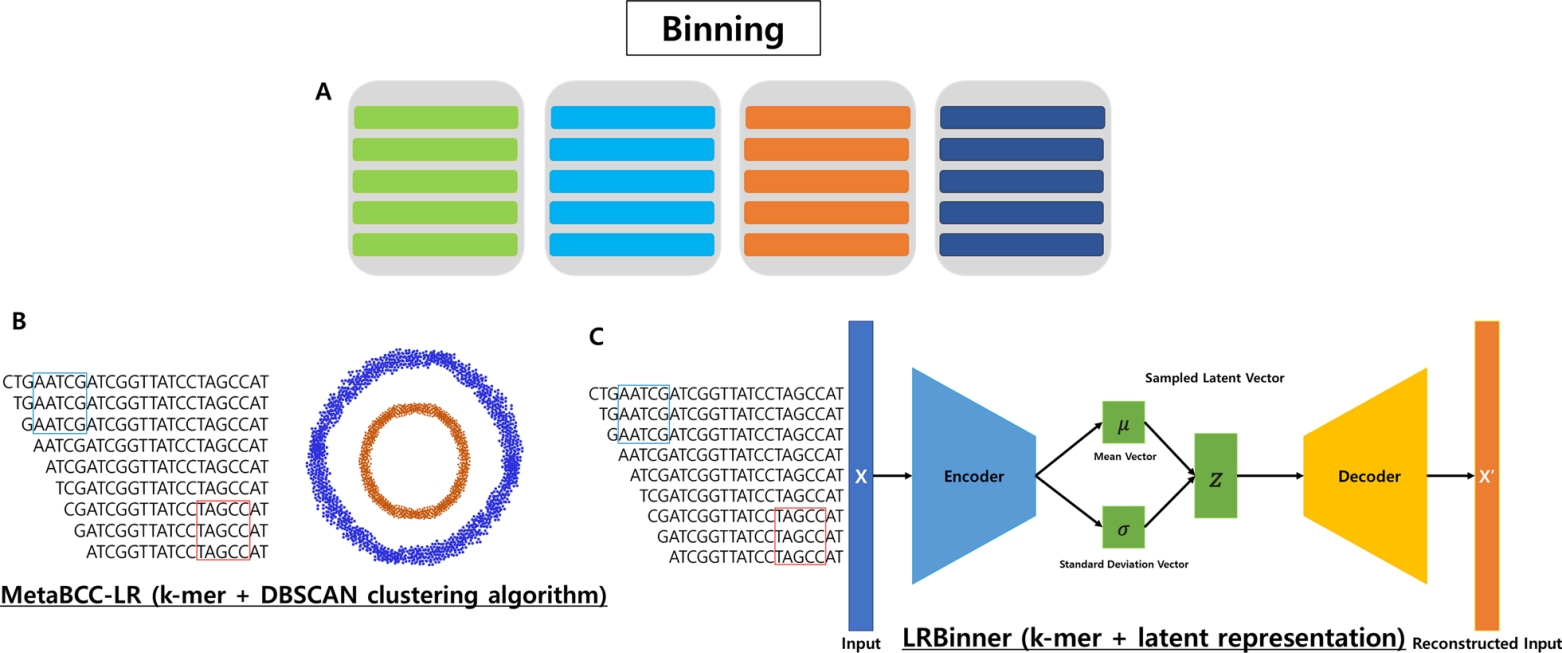
Overview of binning algorithm. **A** Binning is a process in metagenomics where the assembled sequences are clustered or grouped based on their similarities, which helps in reconstructing both known and unknown genomes from complex metagenomic datasets. **B** A visual representation of k-mer coverage and DBSCAN algorithm that forms the key algorithm of MetaBCC-LR. **C** LRBinner enhances sequence binning accuracy by combining composition and coverage information through k-mer profiles and advanced algorithms

After the assembly phase is complete, contigs, which are overlapping segments of short-reads are left. In data science, binning refers to the process of grouping continuous values into smaller sets of “bins” (Fig. 4A). In biology, a similar definition can be applied, where "continuous value" refers to sequences from the reads. During the binning phase, patterns are identified using k-mer profiles that can determine whether two contigs belong to the same genome or not [50]. The patterns are then used to group and link contigs into bins, where each bin is ideally assigned to only one original genome (MAG). There are various ways to separate contigs, including taxonomic assignment, GC content, tetranucleotide composition, and abundance. Taxonomic assignment falls under taxonomic dependent binning, which is a supervised method that compares the sequence to a reference database using aligning algorithms [51]. This method is limited to the reference database, which is oftentimes incomplete. Genome binning is an unsupervised method that clusters contigs into bins using features of the sequences [51]. The two common methods are binning by composition and abundance. Composition based method works on 2 main assumptions: different genomes have distinct sequence features, and a genome has similar sequence features [50]. This method uses % of G/C composition, nucleotide frequency (k-mer frequency), and essential single copy genes as composition features [51]. Usually, longer sequence lengths typically result in better extraction of genome signatures [52]. However, due to increasing sequencing depths and inherent challenges, coverage (abundance) method is becoming more reliable. Differential abundance (coverage) binning methods have 2 main assumptions: sequences have similar abundance level within the same sample if the sequence is from the same genome, and these sequences will display similar abundance level across a multitude of samples [51]. It is possible to combine the two methods (hybrid), especially with longer contigs and newer assembly tools.

Binning of long-reads presents a set of challenges such as a lack of coverage information, which is the information of an average number of reads that is mapped to a position in a reference genome, relatively high error rates, and varying degree of species coverage [53]. When compared to contigs from short-reads, the read length is significantly longer, which requires a unique binning algorithm.

MetaBCC-LR is a long-read binning tool that does not require a reference database to perform the binning. The reads are separated using k-mer coverage histograms and trinucleotide compositions, and statistical models are built for each bin. Then, all the reads are assigned to their respective bins [54]. While MetaBCC-LR employs a multitude of algorithms, obtaining k-mer coverage and using density-based clustering algorithm (DBSCAN) for dimension reduction are notable ones. Instead of estimating the coverage of each read by using all versus all type alignment, using k-mer to break down reads allows for larger metagenomic datasets. DBSCAN is used to reduce dimension and clustering based on both k-mer coverage and trinucleotide composition, which helps with visualization. DBSCAN is a non-parametric density-based clustering algorithm, where the radius of each point of a cluster must contain a certain set of minimum number of points that are closely packed together. The dots that form two circular shapes represent samples or other variables of interest. It is particularly useful since it is density based, allowing it to correctly differentiate two clusters (Fig. 4B). A problem with a number of long-read bin tools is that they either use only composition information, foregoing coverage information, or independent use of composition and coverage information. It can lead to problems such as bins of low abundance species being ignored or bins of non-uniform coverage species being incorrectly categorized [55].

Currently, LRBinner is one of the newest LRS binning tools. Similar to MetaBCC-LR, LRBinner is also a reference free long-read binning tool that can use composition information in addition to the coverage information. LRBinner also utilizes an algorithm that uses distance histograms for the detection and extraction of confident clusters of varying sizes. The tool showcases deep learning algorithms to perform feature aggregation. This comprehensive k-mer representation allows LRBinner to assign sequences more accurately and effectively to their respective bins during the binning process. The VAE (variational autoencoder) is a deep learning algorithm that provides a probabilistic approach to represent observations in a latent space. In the context of LRBinner, the VAE is utilized to learn a latent representation for long-read binning. The general workflow of LRBinner consists of identifying lower dimensional latent representations of both composition and coverage, clustering the latent representations, and obtaining complete clusters. The composition is calculated from normalized k-mer counts. First, the composition and coverage feature vector goes through variational autoencoder (VAE), a type of deep learning that integrates k-mer composition and coverage from metagenomic assemblies. This process is used to obtain low dimensional latent representation. Next, generate a histogram using the distance between a chosen seed point and other remaining points. The peak of the histogram is used to estimate candidate cluster, ultimately resulting in a bin containing the chosen seed point. Lastly, the final bin is obtained by assigning unassigned points to clusters via statistical models such as maximum likelihood. The general structure of VAE can be broken down into 5 parts, the input, encoder, latent space, decoder, and output. Input is fed into the encoder, which uses the mean vector and standard deviation vector to construct the latent space. Latent space is a representation of collection of items that are similar to each other. Then, samples are taken from latent space for the decoder to produce an output that is ideally identical to the original input. When compared to MetaBBC-LR, LRBinner is capable of producing bins with better completeness, lower contamination, better estimation of the number of bins, and overall higher precisions [53] (Fig. 4C).

## Bioinformatic analysis

### Taxonomic annotation

Although there are various bioinformatic tools that can work directly with raw reads after quality control without the need for an assembly phase, data assembly can greatly benefit the process when working de novo without a robust reference database. Taxonomic annotation is the process of classifying the reads using a reference database. Amongst several algorithms used for taxonomic annotation, k-mer based and alignment-based methods were frequently used. Kmer-based method utilizes a short word of length *k* to conduct an exact match [56]. Alignment-based method employs alignment algorithms, such as Bowtie2, to align reads to a reference sequence. In addition to the two different types, there are popular taxonomic classifiers, such as Centrifuge, which uses Burrows-Wheeler transform. Both SRS and LRS have the capacity to provide taxonomic profiles at the species level. Unlike amplicon sequencing, which targets only the hypervariable region, SRS and LRS sequence the entire sample and obtain a higher volume of data. Even in SRS, a relatively unbiased representation of species within the sample can be extracted. This advantage is further pronounced in LRS, where species with abundances as low as 0.1% can be detected with high accuracy. Taxonomic profiles of species are derived during the taxonomic annotation process [57]. To achieve resolution down to the species level, thorough consideration and validation of LRS data are essential, along with the integration of various techniques. These include in situ hybridization for visualizing specific microbial taxa within a sample, quantitative PCR designed to target particular microbial species, traditional cultivation and isolation methods focused on specific microbial species, and whole-genome sequencing of isolated strains [58–61]. The integration of these diverse approaches ensures a comprehensive and accurate characterization of microbial communities.

As discussed in earlier sections, long-reads and short-reads require different approaches including taxonomic annotation. Although conventional tools like Kraken2, Centrifuge, and MetaPhlAn are applicable to both, it is important to select appropriate tools for the type of data, and using inappropriate tools with long-read data may result in several drawbacks. For example, heavy filtering is required to achieve acceptable precision, and even then, the results may yield high false positives, especially in lower abundance regions, and inaccurate abundance estimates [62]. Therefore, using tools that are specifically designed or modified for LRS can produce more reliable results without requiring heavy filtering.

There are two types of algorithms used for the alignment, nucleotide, or protein (translation) alignment methods, on top of the general lowest common ancestor (LCA) shared among the tools. Nucleotide or translation alignment simply refers to whether the alignment relies on each nucleotide or codon for amino acids when comparing against a database. Depending on the sample employed, nucleotide or translation alignment should be selected, for example, DNA database for environmental samples are relatively small and this problem can be mitigated by using translation algorithm. Many taxonomic annotation tools use a naïve algorithm, but a few adjustments must be made for long-read. First, establish segments of reads where alignments accumulate as “conserved genes”. Second, apply naïve LCA to each conserved gene. Lastly, the LCA is used to identify the placement of these reads. This is a summary of the complex algorithm used in taxonomic annotation tools for long-reads. Each long-read taxonomic annotation tool has pros and cons depending on the type of sample being studied (e.g., environmental/anatomical), the type of database used, and available resources. It is important to carefully select the appropriate tool for a specific analysis to achieve reliable results.

Popular tools that utilize translation alignment are MMseq2 and MEGAN-LR. MEGAN-LR is one of the earliest developed tools for long-read while MMseq2 is a newer addition [63]. MEGAN (MEtaGenome Analyzer), developed in 2007, allows large metagenomic datasets to be analyzed [64]. MEGAN6, the newest version, is an all-inclusive toolbox that can perform taxonomic analysis, functional analysis, various visualizations, and metadata, and it was this version that MEGAN-LR was built from. MEGAN-LR can perform translation alignment, which converts nucleotide sequence into a protein, or in this case, aligns nucleotide sequence to the protein reference database.

While MEGAN-LR is compatible with a multitude of tools with translation alignment functions, it is commonly paired with DIAMOND. MEGAN-LR employs interval union LCA algorithm and other features to assign reads to taxa. Interval union LCA works in two steps. First, the reads are fragmented into smaller units known as intervals. This segmentation facilitates the alignment of all read-associated data, ensuring that alignments commence and conclude precisely at the boundaries of these intervals. An alignment is considered significant within an interval if its score falls within the default threshold of 10%. Next, all intervals containing significant alignment associated to a taxon are marked. The taxonomic nodes are then computed in a post order transversal, merging any overlapping intervals along the way. Then the total alignment is computed by placing the read on the taxon [65].

MMseqs2 (Many-against-Many searching) follows 3 steps: k-mer match stage, vectorized ungapped alignment, and gapped (Smith-Waterman) alignment [66]. To perform taxonomic annotation, all protein fragments from long-reads are extracted, extracted protein fragments are filtered and the filtered protein sequences are aligned to a reference database. Finally, the novel LCA algorithm is used to assign reads to aligned sequences [66].

The other method for taxonomic annotation, nucleotide alignment, is represented by tools such as BugSeq-v2 and MEGAN-LR. Among these tools, MEGAN-LR is one of the best tools currently available. It can handle nucleotide alignment using a similar approach to protein alignment, but the reference database is changed from DIAMOND to minimap2 [67]. Bugseq-v2 is a pipeline with an online service, consisting of 5 distinct steps. First, quality control of reads is done by fastp, Next, the reads are mapped with minimap2 (alignment). The alignments are then reassigned using Bayesian statistical framework. Lastly, the LCA is calculated for the reassigned reads and used as an input for Recentrifuge, a tool that allows researchers to perform comparative analysis of multiple metagenomic samples [68]. MEGAN-LR, MMseqs2, and BugSeq-v2 use alignment based and a variation of LCA algorithm as a key player in performing taxonomic annotation.

Aside from the two main groups of taxonomic classifiers, there are also popular classifiers that use different algorithms. One of these is CDKAM (Classification tool using Discriminative K-mers and Approximate Matching), developed to complement third generation sequencing technologies. CDKAM uses approximate matching to search k-mers, that happens in two distinct stages of quick mapping and dynamic programming [69]. It utilizes discriminative k-mer and approximate matching algorithm to perform taxonomic annotation. For discriminative k-mer, first, a reference genome and taxonomy information are downloaded to create database. Next, the k-mer of all strains are collected. Then, genus level is assigned to overlapping k-mers if two or more species share it. After the assignment of genus, k-mers of all species included in the database are combined and repeating or redundant k-mers are removed to produce discriminative k-mers. The discriminative k-mer represents the genomic region unique to each species. CDKAM uses an approximate matching method that is more lenient, allowing for replacements and indels to be included in the match. This allows CDKAM to deal with a relatively higher error rate of LRS while maintaining high computing speed. The program increases the chance of detecting the query sequence within the database [69].

**Fig. 5:**
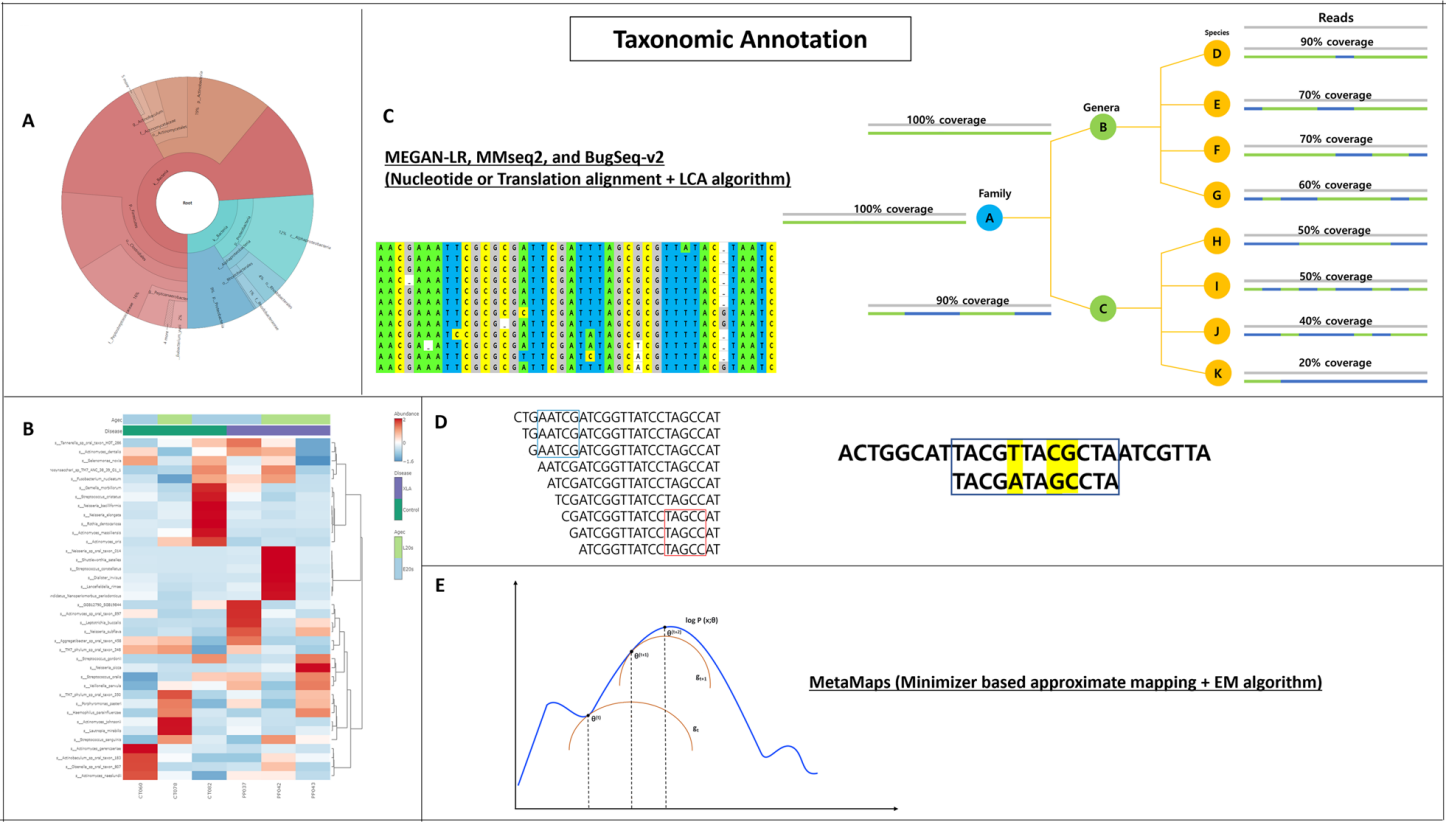
Overview of  Taxonomic Annotation Algorithm. **A** The Krona chart represents hierarchical data, which can be visualized as a multi-layered pie chart and is useful for displaying various levels of taxonomy and their corresponding abundances simultaneously. The pie chart presents all NCBI taxonomy levels, from superkingdom to family, using a blend of radial and spatial display, along with parametric colors and zoom options. **B** Heatmap with hierarchical clustering is one of the more common visualizations of the difference in species abundance. Hierarchical clustering on selected parameters is applied to both rows and columns. Blocks with similar clustering are positioned together, and a color scheme is then applied corresponding to the parameters. **C** Nucleotide or translation alignment uses nucleotides or amino acid codons to search the database. The resulting similarity or dissimilarity can be used to draw conclusions about the relationship between species. Similarities can be indicative of a common ancestor, while mismatches may signify mutations in the form of indels or point mutations. For taxonomic annotation, the LCA algorithm and its variations are commonly employed to determine the taxonomic identity of query sequences based on their similarity to known sequences in the database. The figure depicts eight species, D to K, divided into two genera, B and C, which belong to family A. The read is aligned to the protein sequence from the database, represented in species D to K. The alignment percentage ranges from 90 to 20%. Nodes A and B have read coverage of 100%, while node C has read coverage of 90%. The read is placed on the lowest taxonomic node with ≥ 80% read coverage, which is D. If node D or any other lower taxonomic node has read coverage of 80% or higher, then node B will be chosen. **D** CDKAM utilizes discriminative k-mer and approximates matching algorithm to perform taxonomic annotation. The left image depicts a simplistic view of the k-mer (5-mer) search. The right image depicts approximate matching where the key sequence does not have to be identical but allows mutation or variation. Despite having 3 nucleotide mismatches, the algorithm identifies it as a match. A threshold for approximate matching can be adjusted. **E** MetaMaps employs minimizer-based approximate mapping and the EM algorithm for taxonomic annotation. First, minimizer-based approximate mapping is used to swiftly generate potential mapping location for each long read. Next, all mapping locations are given a score using a probability model, and EM algorithm estimates the overall sample composition. EM algorithm is comprised of two steps: the E-step or estimation step and the M-step or maximization step. The E-step computes missing or latent variables, and the M-step optimizes the parameter to best fit the data. The graph starts with the initial parameter θ^(t)^. E-step constructs the function g_t_ to define the lower bound of the function log P (*x;θ*). The maximum of function g_t_ is θ^(t+1)^ and is computed during M-step. The next E-step defines the new lower bound as function g_t+1,_ and new M-step computes new maximization at θ^(t+2)^. EM steps terminate when parameter estimation converges or reaches maximum iteration

MetaMaps is one of the first tools specifically designed to handle long-reads and utilize approximate mapping with probabilistic scoring methods. MetaMaps analyzes the reads in two steps. Step one, minimizer based approximate mapping method is used to produce potential mapping locations. Step two, a unique statistical model is used to give probabilistic scores to each potential mapping location, where expectation maximization (EM) algorithm is used to estimate the sample microbial composition. Being one of the first long-read taxonomic classifiers, MetaMaps offers three advantages. First, the approximate mapping method allows MetaMaps to regulate the mapping location of each read, and estimate alignment identities and quality of mapping. Second, MetaMaps is resilient against large contaminant genomes. Third, the approximate mapping method is faster than alignment-based methods [70]. Taxonomic annotation can be presented in various visualizations, such as the Krona chart, heatmap, and other graphical representations, to depict the taxonomic composition and relative abundances of different microbial taxa within a metagenomic sample. The general overview of algorithms used by each tool can be seen in Fig. 5 and Table 1 providing a simple representation of a few algorithms employed by each tool. However, the optimal choice of classifier may vary depending on the type and complexity of the sample being analyzed. The byproduct of taxonomic classification is the creation of an abundance profile, which is the estimate of the number of microbes belonging to each species or genus, depending on the customization. While binning tools such as MetaBCC-LR and LRBinner that use coverage provide abundance and composition information, it is during the taxonomic classification stage where bins are labeled and can be better represented visually. When only binning results are obtained, the next essential step involves converting these results into a taxa profile. Subsequently, upon obtaining the abundance profile, the resulting data can be used to conduct differential abundance analysis. This process involves evaluating differences in taxa abundance (such as species or genus) between various samples. The analysis can be conducted using the R software.

**Table 1:** Taxonomic classifiers used for long reads

Name	Algorithm	Reference database	Developed year	LRS affinity
MEGAN-LR [65]	Translation + LCA algorithm	NCBI nt	2018	ONT
	Nucleotide + LCA algorithm	NCBI nt		ONT
MMseq2 [66]	Translation + LCA algorithm	NCBI nt	2017	PacBio
BugSeq-v2 [68]	Nucleotide + LCA algorithm	NCBI nt	2021	ONT
CDKAM [69]	Approximate matching + kmer	NCBI nt	2019	ONT
MetaMaps [70]	Approximate mapping + EM	Miniseq + H	2016	PacBio

In 16S rRNA sequencing, sequences are grouped into Operational Taxonomic Units (OTUs) based on 97% similarity, condensing millions of reads into OTUs and reducing computational load [71, 72]. In contrast to LRS or SRS, 16S sequencing employs unified tools like QIIME2 and MOTHUR. It commonly uses annotated reference sequence databases, such as SILVA, eliminating assembly needs. QIIME2, with BLAST + and VSEARCH, creates a taxonomy consensus classifier, outperforming the original QIIME [73]. Various forms of amplicon sequencing, including ITS sequencing, 18S sequencing, and gyrB sequencing, serve different purposes based on the sample being studied. 16S is instrumental in studying prokaryotes and detecting bacteria and archaea prevalent in microbiomes, especially in living organisms. ITS is optimal for studying the molecular ecology of fungi, while 18 s is used in the study of fungi and protists [74, 75]. QIIME2, mother, or ITS can be adapted as assembly tools for 18S and ITS sequencing, utilizing appropriate databases such as SILVA for 18S rRNA or UNITE database for fungi [76, 77]. Databases for the gyrB gene may not be as extensive or standardized as those for 16S rRNA. However, resources like the National Center for Biotechnology Information (NCBI) offer some sequences for gyrB, and the BLAST assembly tool can be applied.

### Functional annotation

Functional annotation in metagenomics seeks to identify the metabolic and biological pathways of microorganisms present within the sample, providing insights into their potential activities and functional roles in the environment. To achieve this, the identification of protein-coding regions through gene prediction is crucial in metagenomics, but it is more challenging than in isolated genomes due to various reasons.

Currently, there is a scarcity of specialized functional annotation tools tailored for long-read metagenomics. Nevertheless, various tools have been extensively employed in studies utilizing long-read metagenomics data. Similar to assembly and taxonomic profiling, LRS has demonstrated its capability to enhance functional analysis by significantly increasing the proportion of assigned functional annotations compared to short-read sequencing methods. Some of the popular tools include Eggnog-mapper, MEGAN-LR, MetaErg, and MetaWRAP.

**Fig. 6:**
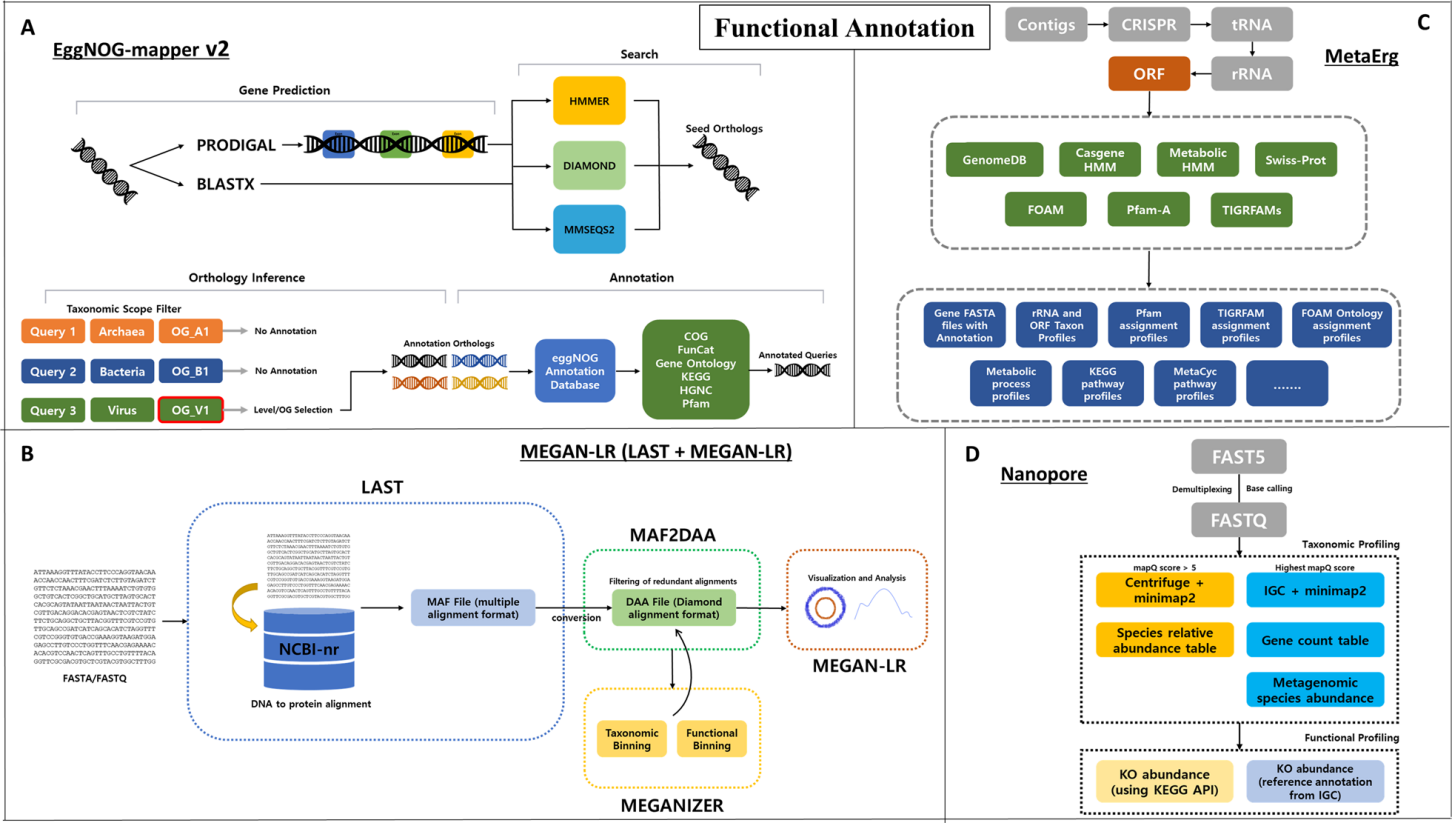
Overview of workflow of functional annotation. Functional annotation utilizes data from previous steps to identify genes and maps them against databases, elucidating the functions of each gene and the respective host microbe. **A** The workflow of EggNOG-mapper v2 consists of gene prediction, search, orthology inference, and annotation stages. Gene prediction uses assembled contigs as input for Prodigal. Search stage aligns the input read against HMMER, DIAMOND, and MMseqs2. During orthology inference, a taxonomic scope filter is applied to get the desired orthologs. Lastly, in the annotation stage, annotated orthologs are put through eggNOG annotation database and other annotation tools, resulting in annotated ortholog. **B** MEGAN-LR starts by aligning the input reads against NCBI-nr, DNA-to-protein database using the LAST alignment tool. The LAST tool outputs a MAF file which is converted into DAA file. The DAA file is taken by Meganizer to perform taxonomic and functional binning, and the outputs are appended back into the DAA file. The newly appended DAA file is then opened in MEGAN-LR for visualization and analysis. **C** MetaErg uses assembled contigs as input and identifies CRISPR region, and non-coding regions, which include tRNA and rRNA. Prodigal uses the outcome to predict the protein coding region or ORF. The predicted ORFs are run through various functional categories, similarity search, and database such as GenomeDB, Casgene HMM, Metabolic HMM, Swiss-Prot, FOAM, Pfam-A, TIGRFAMs, etc. Once functional annotation is complete, output and visualization can be returned in various formats. **D** Nanopore (pipeline) starts by converting fast5 data into fastq files through base calling and demultiplexing. The taxonomic annotation takes fastq files and annotates them using two different tools. The first method uses Centrifuge to perform taxonomic binning and remove erroneous taxonomic assignments using minimap2. Sequences with mapQ score of 5 or higher are kept. The second method uses IGC and minimap2 and only sequence with highest mapQ score is kept. The gene count table is constructed by counting the number of sequences mapped by ONT reads. Using the mean value of the 50 most connected genes from the gene count table, metagenomic species abundance is estimated. For functional annotation, taxonomic results from Centrifuge utilize the KEGG API to retrieve KO content, while those from IGC use the IGC reference to obtain KO content

EggNOG-mapper v2 is the updated version of the original EggNOG-mapper, with improvements in annotation coverage, program capability, and overall performance [78]. EggNOG-mapper v2 consists of four distinct stages. First, the prediction of Open Reading Frames (ORFs) or proteins is accomplished by using the assembled contigs with the help of a widely used tool called Prodigal, which scans the input contigs and identifies potential ORFs based on certain statistical models and sequence characteristics. Second, the predicted ORFs are searched via HMMER3, Diamond, or MMseqs2 against eggNOG and protein databases or HMM similarity search, resulting in seed orthologs. Third, the orthology inference stage generates a list of orthologs depending on the hierarchical level of the taxonomy. Finally, in the annotation stage, the annotation orthologs and domains are processed to the queries, resulting in annotated GFF file and PFAM realignment files [78]. EggNOG-mapper v2 has higher functional annotation precision than the traditional homology searches due to orthology prediction [78] (Fig. 6A).

MEGAN-LR can also be used for functional annotation (or more precisely, functional binning). The length of LRS means there is a high chance of multiple genes being on the same read, and those genes could align to different taxa. This redundancy, where the same gene stretched across different taxa is individually functionally annotated, can be avoided by using the dominance of each alignment. An alignment is dominant over another alignment under 3 conditions. One, if the read covered by alignment B is more than 50% covered by alignment A. Two, if either of the alignment has a higher bit score, a score showing the statistical significance of an alignment. Three, if the same read contains both alignments [65]. During the functional annotation process, functional classes are assigned to alignments that are not dominated by other alignments. The general workflow of MEGAN-LR is as follows. Long-read is put through LAST program that performs DNA to protein alignment and outputs long protein alignment over long-reads in MAF file format. MAF2DAA takes the chunky MAF file and converts it into DAA file. During the conversion, MAF2DAA filters out strongly dominated alignments and removes many redundant alignments. The resulting DAA file is put through Meganizer for taxonomic and functional annotation [65] (Fig. 6B).

MetaErg is “a stand-alone and fully automated metagenomic and metaproteomic data annotation pipeline” [79]. MetaErg can perform feature prediction, functional annotation, and estimation and visualization of quantitative taxonomic and pathway composition. The functional annotation starts by inputting predicted ORF into HMM and DIAMOND-based profile similarity searches. The resulting ORFs are then aligned and searched against different databases (MetaErg accepts external databases or built-in databases that are constructed using publicly available databases such as SILVA, RefSeq. FOAM, casgene.hmm, etc.). The search results are then combined to link query gene with various aspects such as functional categories, KO terms, GO terms, EC numbers, protein domains, metabolic potential and trains [79]. In total, ORF from MetaErg is searched against HMM from Pfam-A, TIGRFAM, FOAM, Metabolic-hmm, and SwissProt. The mapping files derived from these searches are used as input for MinPath, which reconstructs metabolic pathways and infer KEGG and MetaCyc pathways [79] (Fig. 6C).

There is another method of performing functional analysis for reads from ONT. A pipeline was developed in 2021 for data processing and analysis, especially for the estimate of microbial composition and diversity [80]. Within the pipeline (simply named Nanopore), there is a functional analyses command. This analysis relies on the species abundance table to compute KO abundance and KO richness to get functional annotation. To achieve optimal results in functional analyses using this pipeline, it is recommended to utilize the entire pipeline, as the outputs from previous steps are required to proceed (Fig. 6D, Table 2).

**Table 2:** Functional annotation tools and pipelines

Name	Developed year	Database
EggNOG-mapper v2 [78]	2021	eggNOG
Nanopore (pipeline) [90]	2021	KEGG
MetaERG [79]	2019	casgene.hmm FOAM metabolic-hmms Pfam SwissProt SILVA SSU TIGRFAMS GTDBTK RefSeq
MEGAN LR [65]	2018	EC eggNOG InterPro SEED KEGG

## Discussion

Metagenomics is a relatively young field with a shorter history; the term was coined in 1988, and the concept first appeared in 1985, compared to other omics fields such as genomics, which was coined in 1986 but began in the 1970s, and proteomics, coined in 1994 but initiated in 1975. Notably, whole-genome shotgun sequencing, which redefined metagenomics, was introduced in 2004 [81–84]. Microbial studies continue to utilize 16S rRNA sequencing due to its affordability and the availability of a comprehensive reference database. However, the incorporation of LRS into metagenomics has introduced a new technology to the field, unlocking numerous possibilities. LRS has expanded the scope of metagenomic research, enabling in-depth analysis of complex microbial communities and offering valuable insights into the functional potential and taxonomic diversity that were previously challenging to explore. LRS has the potential to bring significant improvements and provide valuable insights in areas where short-read sequencing falls short. Its ability to generate longer and more contiguous reads allows for better characterization of complex genomic regions, resolving repetitive elements, and identifying novel sequences. In theory, LRS holds the potential to overcome many drawbacks of short-read sequencing. However, in practice, LRS has faced several major challenges, including higher costs and a lack of dedicated tools. Despite these initial hurdles, LRS providers, such as ONT and PacBio, have made significant strides in improving accuracy, and the sequencing costs have gradually decreased and are expected to continue doing so. Moreover, more dedicated tools are continually being developed to tackle the challenges of LRS in metagenomics. LRS is expected to play a crucial role not only in metagenomics but also in genomic research, enabling comprehensive and accurate analysis of complex genomes, resolving structural variations, and identifying novel genetic elements. With these advancements, LRS has the potential to significantly contribute to various scientific disciplines and drive important discoveries in the field of biology and beyond. LRS and SRS are not mutually exclusive and can be used to complement one another. SRS offers superior accuracy at a lower sequencing cost while capturing microbial diversity within well-defined regions. LRS, on the other hand, are better at mapping genetic regions with high structural variability and repetitive regions, identifying haplotype information and co-inherited alleles, and detecting taxa with low abundance [85]. By strategically combining these technologies, researchers can leverage the accuracy of short-reads for precise taxonomic identification and functional profiling in specific genomic regions, while utilizing the ability of long reads to elucidate the structural organization and interactions among microbial species [86].

Obtaining species-level resolution using 16S rRNA sequencing is often challenging due to limited variability at the species level. The 16S gene is a commonly used marker for microbial identification, but its sequence conservation within certain bacterial taxa makes it more suitable for genus and family-level classifications. Although BLAST against the NCBI database is an option, it lacks reliability. To improve resolution, utilizing an environment-weighted taxonomy classifier with alternative weight assumptions has been shown to enhance results [87]. To achieve species-level, alternative markers such as the 18S rRNA gene or other markers like gyrB may be considered. The selection of markers depends on the study's specific objectives and the taxonomic group being examined. Each marker has its advantages and limitations. The 18S rRNA is commonly used for eukaryotic microorganisms. It provides higher variability than the 16S and can offer improved resolution at the species level for certain taxa, especially among fungi and protists. The gyrB can exhibit higher variability than the 16S gene, potentially allowing for better discrimination at the species level among certain bacterial groups such as the Bacillus subtilis group and genera like Myxococcus, Corallococcus, and Pyxidicoccus [88, 89]. In the future, researchers may explore alternative markers or use a combination of markers to enhance taxonomic resolution and accuracy in microbiome analysis.

ONT and PacBio, as two leading long-read sequencing platforms, each offer distinct advantages and disadvantages. ONT stands out for its longer read lengths, particularly with their new ultra long-read sequencing, which allows for better resolving complex regions and characterizing large genomic elements. On the other hand, PacBio provides relatively shorter reads but higher accuracy, enabling more precise base calling. While ONT's longer read lengths appear advantageous, both platforms have made significant improvements in accuracy, making the decision less straightforward. The decision between ONT and PacBio for LRS is multifaceted and relies on specific research needs. Various factors, such as the desired read length, sequencing accuracy requirements, the complexity of the genome or metagenomic sample, and the available budget, all play a crucial role in determining the most suitable platform for a given application. For complex and larger cells like eukaryotes with larger genomes, PacBio’s iso-seq and HiFi sequencing can be advantageous due to their better ability to call structural variations accurately. These platforms are particularly useful for applications where precise resolution of genomic rearrangements is crucial. Furthermore, each platform has specialized tools that may perform better for specific analysis tasks, making it essential to assess the compatibility of the available tools with the intended research goals. Besides technical considerations, external factors such as cost, and the availability of the service are vital in determining the most suitable platform. The MinION from ONT is a cost-effective option, making it attractive for researchers with budget constraints. Additionally, familiarity with a particular platform and the availability of support or expertise can influence the decision-making process. Ultimately, researchers need to carefully weigh these factors and tailor their choice of long-read sequencing platform based on the specific requirements of their research project, ensuring the most effective and reliable outcomes.

## Conclusions

Both ONT and PacBio platforms provide valuable insights into the future of long-read sequencing, offering longer reads that can capture low abundance taxa and improved species identification, thereby generating more accurate microbial profiles. As these technologies continue to advance, they hold immense potential to revolutionize our understanding of complex microbial communities and their functional capabilities in diverse environments, contributing significantly to the field of metagenomics and genomic research.

## Data Availability

All data generated or analyzed during this study are included in this published article.

## Abbreviations

CDKAM: Classification tool using discriminative K-mers and approximate matching
DBSCAN: Density-based clustering algorithm
EM: Expectation maximization
LCA: Lowest common ancestor
LRS: Long-read sequencing
MAG: Metagenome assembled genome
MEGAN: MEtaGenome analyzer
MMseqs2: Many-against-many sequence searching
NIH: National Institute of Health
OLC: Overlap layout consensus
ONT: Oxford Nanopore Technologies
ORFs: Open reading frames
PacBio: Pacific Biotechnologies
QC: Quality control
VAE: Variational autoencoder
